# Phenolic profile of selected grape ( *Vitis vinifera* L.) varieties in the course of ripening and *Botrytis cinerea* infections

**DOI:** 10.1002/jsfa.70959

**Published:** 2026-08-05

**Authors:** Kim Marie Umberath, Louis Backmann, Maren Scharfenberger‐Schmeer, Pascal Wegmann‐Herr, Andreas Schieber, Fabian Weber

**Affiliations:** ^1^ Institute of Nutritional and Food Sciences, Molecular Food Technology, Faculty of Agricultural, Nutritional and Engineering Sciences University of Bonn Bonn Germany; ^2^ Institute for Viticulture and Oenology Dienstleistungszentrum Ländlicher Raum (DLR) Rheinpfalz Neustadt Germany; ^3^ Department of Biology, Chemical Plant Ecology Technische Universität Darmstadt Darmstadt Germany; ^4^ Hochschule Kaiserslautern Weincampus Neustadt Neustadt Germany; ^5^ Section of Organic Food Quality University of Kassel Witzenhausen Germany

**Keywords:** *Botrytis cinerea*, grapevine, grey mold, laccase, phenolic profile, *Vitis vinifera*

## Abstract

**BACKGROUND:**

Grapevine phenolic compounds play key roles in plant defense and wine quality. Composition depends on factors such as maturity and infections such as grey mold caused by *Botrytis cinerea*. Grapevines respond to infections by actively modifying their phenolic profile. These changes, along with fungal enzymes such as laccases, influence the overall phenolic composition. As a result of climate change, incidence and severity of infections are increasing, with different varieties being susceptible to varying degrees. This study examines differences in susceptibility and the impact of ripening and *B. cinerea* infection on phenolic profiles in three grape varieties over two vintages.

**RESULTS:**

Heavy rainfall following prolonged drought increased the risk of infection. Occurrence of infection varied by sugar content, with Riesling showing higher susceptibility than Pinot noir. Compared to the non‐infested Pinot gris variety, the profile changes primarily involved hydroxycinnamic acids and methoxylated compounds. Flavonols were the only group not affected at all. A myricetin hexoside, which is usually not synthesized by white cultivars, was detectable in infested Riesling. Pinot gris and Pinot noir were characterized by high levels of trihydroxylated polyphenols. Riesling showed decreasing levels of phenolics in early ripening stages and induction only at advanced infection. For Pinot noir, the opposite development was observed.

**CONCLUSION:**

Modifications in phenolic composition can be dependent on cultivar, *Botrytis* laccase activity and substitution pattern of involved compounds. Trihydroxylated compounds might contribute to resistance. These insights provide a foundation for further targeted studies and grape breeding efforts incorporating phenolic profile as an additional factor for resistance. © 2026 The Author(s). *Journal of the Science of Food and Agriculture* published by John Wiley & Sons Ltd on behalf of Society of Chemical Industry.

## INTRODUCTION

Non‐volatile phenolic compounds represent one of the most abundant and heterogenous groups of plant secondary metabolites. In grapevine (*Vitis vinifera* L.), several phenolic sub‐classes with high structural diversity can be found, such as tannins, stilbenes, hydroxycinnamic acids and flavonoids.^1^ Figure 1 provides an overview of the biosynthesis via the phenylpropanoid and the flavonoid pathway involved in the formation of certain polyphenols in grapevines. The phenolic composition of grapes depends on numerous factors, including variety,^2^, ^3^ vintage^4^ and (micro)climate.^5^, ^6^ The state of maturity significantly impacts the phenolic profile because of biochemical changes during ripening. It is commonly assumed that flavanols and stilbenes account for most of the phenolic content present at véraison,^7^ whereas flavonol and anthocyanin levels increase post‐véraison.^8^ Polyphenols are integral to the plant's defense mechanism, and although some of them are produced constitutively (phytoanticipins),^9^ their levels often change in response to infections (phytoalexins).^8^ Fungal infections such as grey mold generally have adverse effects on the quality and characteristics of the wine produced. The grey mold pathogen *B. cinerea* is a necrotrophic fungus infecting a wide range of hosts.^10^ Infections lead to detrimental effects on grapevines globally,^11^ ranging from sensory alterations like discoloration up to total loss of the harvest.^12^ Unlike the effects of grey mold, infections of ripe berries with *B. cinerea* under particular environmental conditions can improve wine quality and are referred to as noble rot.^12^

**Figure 1:**
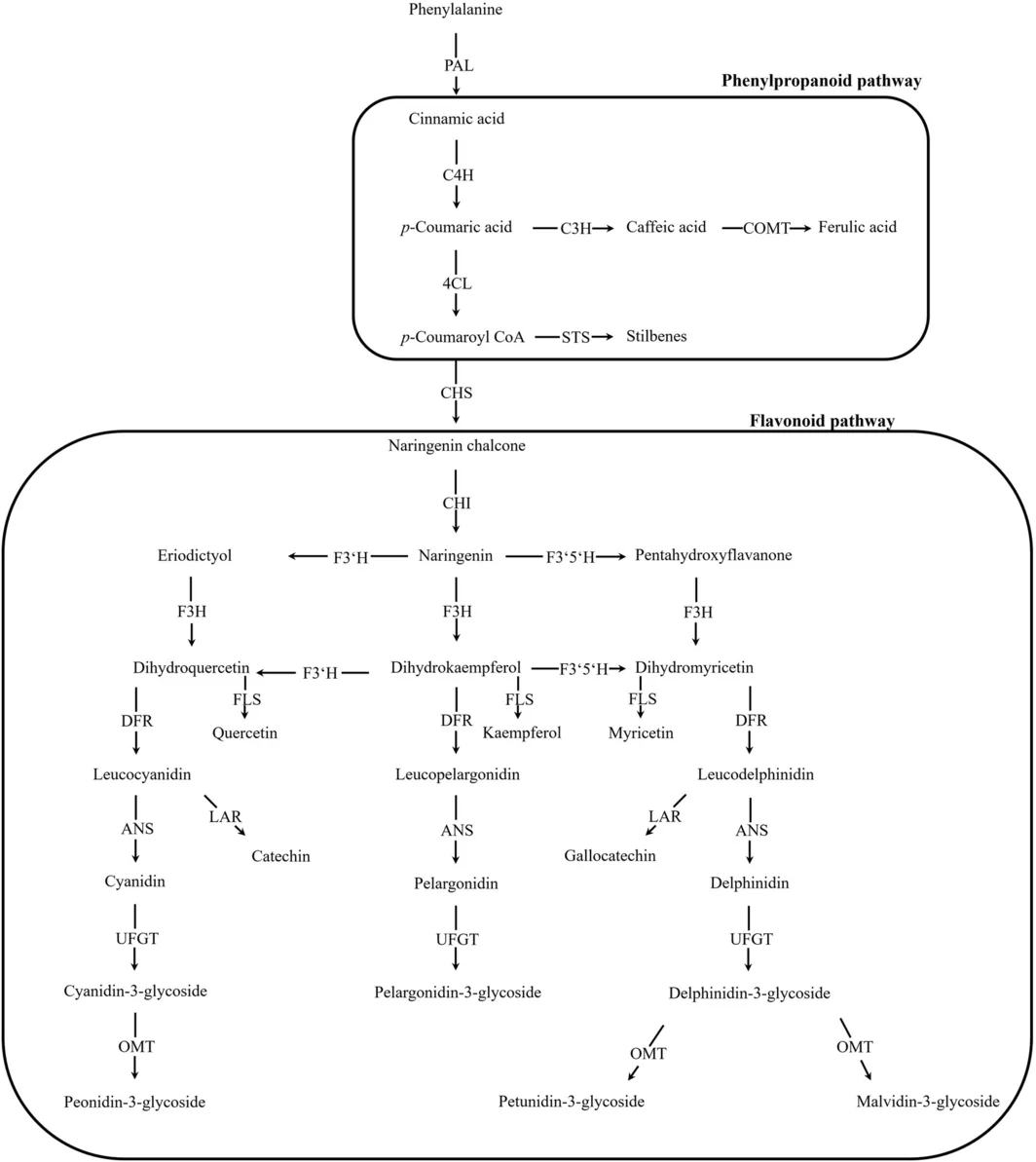
Crucial parts of the phenylpropanoid and flavonoid pathway in grape polyphenol synthesis. ANS, anthocyanidin synthase; 4CL, 4‐coumaroyl‐CoA ligase; C3H, *p*‐coumarate 3‐hydroxylase; C4H, cinnamate 4‐hydroxylase; CHI, chalcone isomerase; CHS, chalcone synthase; COMT, caffeic acid 3‐*O*‐methyltransferase; DFR, dihydroflavonol 4‐reductase; F3H, flavonon 3‐hydroxylase; F3'H, flavonoid 3′‐hydroxylase; F3'5'H, flavonoid 3′,5′‐hydroxylase; FLS, flavonol synthase; LAR, leucoanthocyanidin reductase; OMT, *O*‐methyltransferase; PAL, phenylalanine ammonia lyase; STS, stilbene synthase; UFGT, UDP‐Glc:flavonoid 3‐*O*‐glucosyltransferase. Adapted from Cataldo *et al*.^41^ and Blanco‐Ulate *et al*.^28^

*Botrytis* secretes several enzymes for the alteration of plant cell walls to facilitate the entry of the appressorium.^10^ A key virulence factor of the *Botrytis* secretome is laccase. Laccases (EC 1.10.3.2.) are multicopper enzymes that couple the four‐electron reduction of oxygen to water with the oxidation of a variety of substrates including phenols.^13^ Consequently, highly reactive *o*‐quinones are formed which can undergo further polymerization to brown pigments.^14^ As a result, the antimicrobial properties of polyphenols are altered, although it was shown that laccase‐mediated oxidation products can possess antifungal effects as well.^15^, ^16^ Laccase substrate specificity differs substantially between *Botrytis* strains,^17^ and it has been demonstrated that a high degree of infection does not necessarily correlate with elevated laccase activity.^16^ *Botrytis cinerea* produces multiple laccases, with the secretion of external laccases being significantly induced by phenolic compounds present in grape skin.^18^, ^19^, ^20^ At the same time, the plant modifies polyphenol synthesis as a stress response to the infection. Stilbenes and flavanols are not only produced pre‐véraison, but levels also increase after infections.^20^, ^21^, ^22^, ^23^ Other phenolic groups such as anthocyanins, flavonols and hydroxycinnamic acids are assumed to exclusively decrease as a result of oxidation,^22^, ^24^, ^25^ leading to wines of lower quality.^1^

The incidence and severity of grey mold infections have increased significantly worldwide in recent years because of earlier grape maturation^26^ and an increase in heavy rainfalls,^28^ which raises the need to develop adapted strategies against *Botrytis* infections. Understanding the dynamic reciprocal impact of fungus and grapevine depicts a key challenge here. Although genetic modulations of the flavonoid and phenylpropanoid pathways during noble rot infections have increasingly been examined,^23^, ^28^ research on alterations in phenolic composition during naturally occurring grey mold infections across varieties and vintages is rare.^29^, ^30^ Recent studies on changes in the phenolic profile focused on the classification into the phenolic sub‐classes hydroxycinnamic acids, stilbenes and flavonoids. These sub‐classes vary significantly in their degree of hydroxylation, where substitution patterns of the phenolic rings may be similar even between otherwise distinct groups. However, because the substitution patterns have a decisive influence on the susceptibility to oxidation of polyphenols, this aspect appears to be important for the discussion. The objective of this study is to elaborate potential phenolic profile modulations in the course of ripening and *B. cinerea* infections with respect to conventional phenolic sub classes, but also the different substitution patterns are taken into account. The impacts of weather conditions on *Botrytis* infections are considered as they have been described to affect water uptake and alongside phenolic concentrations in grapes.^27^, ^45^ For this purpose, monitoring of the phenolic composition, *B. cinerea* biomass and laccase activity of three grape varieties (Riesling, Pinot gris and Pinot noir) over the course of two consecutive vintages in the Palatinate region of Germany was conducted. The overall goal is to enhance the current understanding of host responses and *Botrytis* pathology, providing a foundation for future targeted studies.

## MATERIALS AND METHODS

### Chemicals and reagents

Buffers contained sodium acetate (purity ≥ 98.5%; Carl Roth, Karlsruhe, Germany) and glacial acetic acid (purity ≥ 99.7%; VWR Chemicals, Radnor, PA, USA). Polyvinylpolypyrrolidone (Smartvin®) for removal of inhibitory compounds prior to laccase activity assessment was obtained from Enologica Vason (S.p.A, San Pietro in Cariano, Italy). Syringaldazine (purity 99%; Sigma‐Aldrich, Saint Louis, MO, USA) was used as the substrate for determination of laccase activity. Ethanol for enhanced solubility of syringaldazine was obtained in analytical grade (purity 99.8%; Thermo Fisher Scientific, Waltham, MA, USA). Methanol (purity ≥ 99.9%; VWR Chemicals) was used for polyphenol extraction. Further reagents for the determination of skin tannins comprised triethanolamine (purity ≥ 98%), iron(III) chloride (purity 97%), maleic and tartaric acids (purity ≥ 98% each), and urea (purity ≥ 98%) (each from Alfa Aesar GmbH & Co. KG, Karlsruhe Germany), as well as hydrochloric acid (37%) and potassium disulfite (purity ≥ 96%) by VWR International GmbH (Darmstadt, Germany). Bovine serum albumin fraction V (purity ≥ 98%; Carl Roth GmbH & Co. KG, Karlsruhe, Germany), sodium hydroxide (purity ≥ 98%; Honeywell Inc., Offenbach, Germany) and sodium chloride (purity 100%; Labochem Int., Athen, Greece) were used as well. Formic acid (purity ≥ 98%; Sigma‐Aldrich), acetonitrile (purity ≥ 99.9%; Thermo Fisher Scientific) and deionized water (Merck, Darmstadt, Germany) were used as solvents for ultrahigh performance liquid chromatography–electrospray ionization tandem mass spectrometry (UHPLC‐ESI‐MS^n^) analysis. For external calibration, ferulic acid (purity ≥ 99%) and *p*‐coumaric acid (purity ≥ 98%) (Thermo Scientific, Kandel, Germany), (+)‐catechin hydrate (purity ≥ 98) and quercetin‐3‐β‐d‐glucoside (Q3G) (purity ≥ 90%) (Sigma‐Aldrich), caffeic acid (purity ≥ 95%; Merck), resveratrol (purity 99.94%; Apollo Scientific, Bredburry, UK) and malvidin‐3‐*O*‐glucoside chloride (M3G) (purity > 85%; PhytoPlan GmbH, Heidelberg, Germany) were used.

### Meteorological data

Data regarding total rainfall and average hours of sunshine for the respective winegrowing area near Neustadt an der Weinstraße in the Palatinate region in Germany were extracted from the database of the DWD (Deutscher Wetterdienst, Offenbach, Germany).

### Grape samples

Three grape varieties were sampled over two vintages (2022 and 2023) near Neustadt an der Weinstraße. Vineyard coordinates were: Riesling (RS) 49°38′31.73′′N 8°17′98.75′′E, Pinot gris (PG) 49°22′59.3′′N 8°10′42.6′′E and Pinot noir (PN) 49°22′26.3′′N 8°11′21.9′′E. All sites lie at an elevation of 140 m, oriented north–south and surrounded by other vines on a plain area. Sampling started before véraison and continued until late harvest. The bunch sampling protocol followed Nadal.^31^ To minimize inter‐vine variation, bunches were collected from randomly selected vines, including sunny and shaded sides of each row. At each date, 50 bunches per variety were harvested, and berries were randomly taken from the top, middle, and bottom of each bunch, resulting in 3 × 100 berries per variety as field triplicates. Fresh aliquots were transported cooled to the laboratory, frozen at −80 °C, and stored at −20 °C. Frozen berries were destemmed and seeds removed to avoid seed tannin extraction. Dry mass was determined by lyophilization, and fresh weight was corrected accordingly. Basic grape juice parameters were measured using Fourier transform infrared (FTIR) (Wine Scan™ FT120 Basic; FOSS GmbH, Hamburg, Germany).

### Real‐time PCR analysis of *B. cinerea* biomass

Real‐time quantitative PCR (qPCR) analysis of all grape samples was conducted according to Backmann *et al*.^32^ to quantify *Botrytis* biomass (BM). In brief, homogenized berries were extracted using RED Extract Plant PCR kit (Merck). A standard curve was obtained by serial dilution of a *B. cinerea* spore suspension. qPCR measurements were performed with the following protocol: hold stage at 95 °C for 3 min, two‐step PCR at 95 °C for 15 s and 60 °C for 30 s (40 cycles), melting curve at 95 °C for 15 s, 50 °C for 60 s, and 95 °C for 1 s. The following primers targeting the ribosomal region between 28S and 18S were used for amplification: Bc10nt_3R: 5′‐GGA GCA ACA ATT CGC ATT TCA AAC ATG CTG ‐3′ and Bc10nt_3F: 5′‐ GCT GTA ATT TCA ATG TGC AGA ATC CTG TCC CCG GT‐3′.

### Analysis of laccase activity

Laccase activity (LA) was analyzed following Porep *et al*.^33^ with minor modifications. Homogenized berries were pressed through synthetic fiber tissue to obtain must. For removal of interfering compounds, 5 mL of must and 7 mL of 0.1 m sodium acetate buffer (pH 5) were stirred with 0.5 g of polyvinylpolypyrrolidone for 10 min, then centrifuged and membrane‐filtered (0.45 μm). LA was quantified using the syringaldazine assay according to Zimdars *et al*.^17^ with a FLUOstar Omega microplate reader (BMG Labtech, Ortenberg, Germany). A 1.5 mm syringaldazine stock solution (ethanol) was diluted in sodium acetate buffer (46 mm, pH 5). Reactions contained 100 μL of must and 200 μL of substrate and were run at 25 °C, measuring absorbance at 595 nm for 3 h. The must served as blank. Enzyme activity was calculated using the syringaldazine extinction coefficient, with one unit (U_Lacc_) defined as the oxidation of 1 μmol of syringaldazine per minute.^34^

### Phenolic composition

#### Extraction of phenolic compounds

The destemmed seedless berries were homogenized, and approximately 6 g mash was extracted using 20 mL methanol/water (70/30 v/v) with the addition of 0.1% formic acid. Mash and extraction solution were vortexed and treated with ultrasound (USC600D; VWR International GmbH) for 10 min. Subsequently, the samples were centrifuged at 4 °C for 10 min at 16 200 *g* (Heraeus Megafuge 40R; Thermo Fisher Scientific). Following decanting, extraction was repeated twice using 10 mL of extraction solution. Extracts were combined, made up to 50 mL and stored at −80 °C until analysis.

#### Quantification of skin tannins

Skin tannins were quantified via the protein precipitation assay by Harbertson *et al*.^35^, ^36^ with slight adjustments. Extracts were not diluted before analysis. After protein precipitation, samples were bleached using bisulfite and resuspended in a reformulated buffer (urea 8.3 m, 5% triethanolamine, pH 7 adjusted with HCl).^37^ Absorbance was measured at 510 nm (Spectrophotometer V‐730; Jasco GmbH, Pfungstadt, Germany) before and after addition of 125 μL of ferric chloride solution. Tannins were expressed as catechin equivalents using an external calibration curve.

#### Identification and quantification of low molecular phenolic compounds

Extracts were prepared as described in section 2.6.1 were filtered through 0.45 μm membranes. UHPLC‐ESI‐MS^n^ analysis was carried out on an Acquity UPLC I‐Class system (Waters, Milford, MA, USA) with a cooled autosampler (10 °C), a 40 °C column oven and diode array detection (190–800 nm). Phenolics were separated on a Waters Acquity HSS‐T3 RP18 column (150 × 2.1 mm, 1.8 μm) using a 0.5 mL min^−1^ gradient of formic acid in water (A) and formic acid in acetonitrile (B; 3% for anthocyanins, 0.1% v/v for other polyphenols): 0 min, 5% B; 8 min, 10% B; 25 min, 25% B; 26–28 min, 100% B; 30–31 min, 5% B. The system was coupled to an LTQ XL ion trap mass spectrometer (Thermo Fisher Scientific) with ESI in negative or positive mode. Anthocyanins were analyzed in positive mode. Negative‐mode settings: 325 °C capillary temperature, −21 V capillary voltage, 3 kV source voltage, 100 μA current, −60 V tube lens. Positive‐mode settings: 325 °C capillary temperature, 40 V capillary voltage, 5 kV source voltage, 100 μA current, 70 V tube lens. Full‐scan spectra (*m/z* = 150–2000) were used for identification based on fragmentation and literature data. Quantification employed single ion monitoring with external calibration. Anthocyanins were expressed as M3G equivalents, flavanols as C equivalents, flavonols as Q3G equivalents and stilbenes as resveratrol equivalents. The three hydroxycinnamic acid tartrate esters (HCA) were quantified using their corresponding free acids. Limits of quantification corresponded to the lowest calibration point; limits of detection were based on a signal‐to‐noise ratio of three.

### Statistical analysis

For statistical analysis, XLSTAT, version 2019.1.1 (AddinSoft Technologies, Paris, France) was used. Principal component analysis (PCA) and analysis of variance with a defined significance level of *P* < 0.05 was performed in the case of variance homogeneity. Pearson correlation coefficients with a significance level of *P* < 0.05 were calculated to determine correlations between variables. Normalization of total phenolic concentrations was achieved by *z*‐transformation.

## RESULTS

### Meteorological data, maturation and *B. cinerea* infection process

Figure 2 presents total precipitation, the 10‐year average, and sampling dates for both vintages. BBCH 81 (onset of ripening) served as the reference point. In 2022, substantial rainfall occurred around flowering, followed by extended dry periods during early ripening, with totals below the 10‐year average. Heavy rainfall began in late August, approximately 2 weeks after BBCH 81, and continued until harvest. By contrast, precipitation in 2023 was more evenly distributed. Total rainfall exceeded the 10‐year average before BBCH 81, whereas post‐véraison rainfall remained moderate, which did not create conditions conducive to *B. cinerea* infections. Precipitation was above the 10‐year average for this week but less than in 2022.

**Figure 2:**
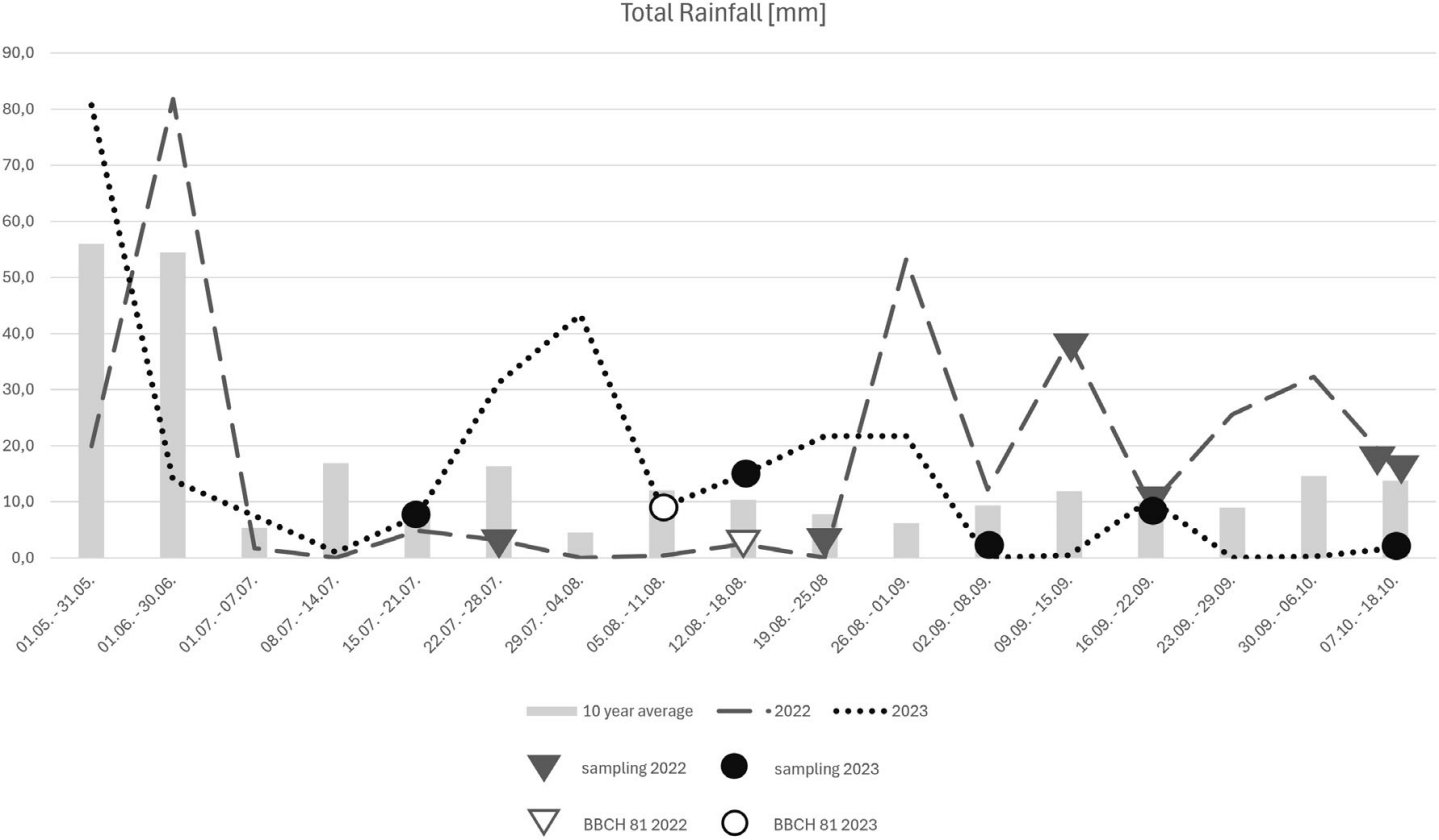
Total rainfall in both vintages and 10‐year average. Sampling points and BBCH 81 are marked. BBCH value determined for Riesling.

FTIR analysis of the musts indicated faster ripening of all varieties in 2022 compared to 2023, although BBCH 81 occurred only 5 days apart (see Supporting information, Table S1). As expected, at technological maturity, RS comprised the highest titratable acidity (TA) content and lowest total soluble solids (TSS) in both vintages.

Table 1 summarizes BM and LA. PG was not infested in either vintage. PN was infested in 2022, with initial BM detected at sampling point 5 (56 days after BBCH 81; TSS > 21.6 °Bx) with low BM, increasing significantly until harvest. LA appeared simultaneously and rose accordingly. RS was infested with *B. cinerea* in both vintages. In 2022, infection began at sampling point 3 (24 days after BBCH 81; TSS 17.8 °Bx) with low BM and no LA. At the following sampling point, LA was measurable with increasing BM (TSS 18.8 °Bx). Both parameters increased markedly by harvest. In 2023, only BM was quantifiable, starting at sampling point 3 (36 days after BBCH 81; TSS 16.2 °Bx). No correlation between BM and LA was evident, consistent with the findings reported by Umberath *et al*.^18^ The present results confirm that LA is not suitable for early *Botrytis* detection.^31^

**Table 1:** Infection parameters in both vintages

	2022				2023	
Sampling point	D_81_	Biomass (spores)	Laccase activity (mU mL^−1)^	D_81_	Biomass (spores)	Laccase activity (mU mL^−1^)
1	−20	n.d.	n.d.	−23	n.d.	n.d.
2	9	n.d.	n.d.	4	n.d.	n.d.
3	24	RS: 717 ± 920	n.d.	36	RS: 427 ± 27	n.d.
4	36	RS: 6477 ± 6146	RS: 0,058 ± 0,017	40	RS: 233 ± 71	n.d.
5	56	RS: 140219 ± 84 967	RS: 2,8 ± 0,53	61	RS: 37569 ± 18 851	n.d.
		PN: 43 ± 52	PN: 0,04 ± 0,008			
6	63	PN: 43120 ± 31 607	PN: 0,25 ± 0,014			

*Note:* No sampling point 6 was conducted for Riesling in 2022 and for neither variety in 2023. D_81_, days prior to or after BBCH 81; RS, Riesling; PN, Pinot noir; n.d., not detected. BBCH value determined for Riesling. Results are expressed as the mean ± SD of triplicates.

### Composition of phenolic sub‐classes throughout ripening

Figure 3 summarizes the UHPLC‐ESI‐MS^n^ results for the phenolic composition of RS, PG and PN, as well as skin tannins analyzed via the protein precipitation assay, normalized to their respective maxima. Concentration for the individual compounds can be found in Tables S2‐S4 (Supporting information). Sampling 1 prior to BBCH 81 marks the maximum concentration in most cases, consistent with previous studies.^2^ Most phenolics are synthesized pre‐véraison to constitutively prevent microbial infections. Exceptions are flavonols and anthocyanins, for which genetic expression is induced after véraison.^38^ During ripening, variety‐specific differences emerged for most phenolic classes.^39^ Flavonols constitute an exception here because the concentration increased steadily in all varieties until reaching a plateau around harvest, likely a result of continuous sunshine,^40^, ^41^ which occurred in both vintages (see Supporting information, Fig. S1). Therefore, the assumption that flavonols steadily increase during ripening^42^ can be confirmed by these data. No infection‐related changes were evident for this group. Myricetin hexoside was tentatively identified in RS, which was not anticipated, because white grape varieties typically do not synthesize it. It was first reported in white grapes by Carbajal‐Ida *et al*.^22^ during noble rot.

**Figure 3:**
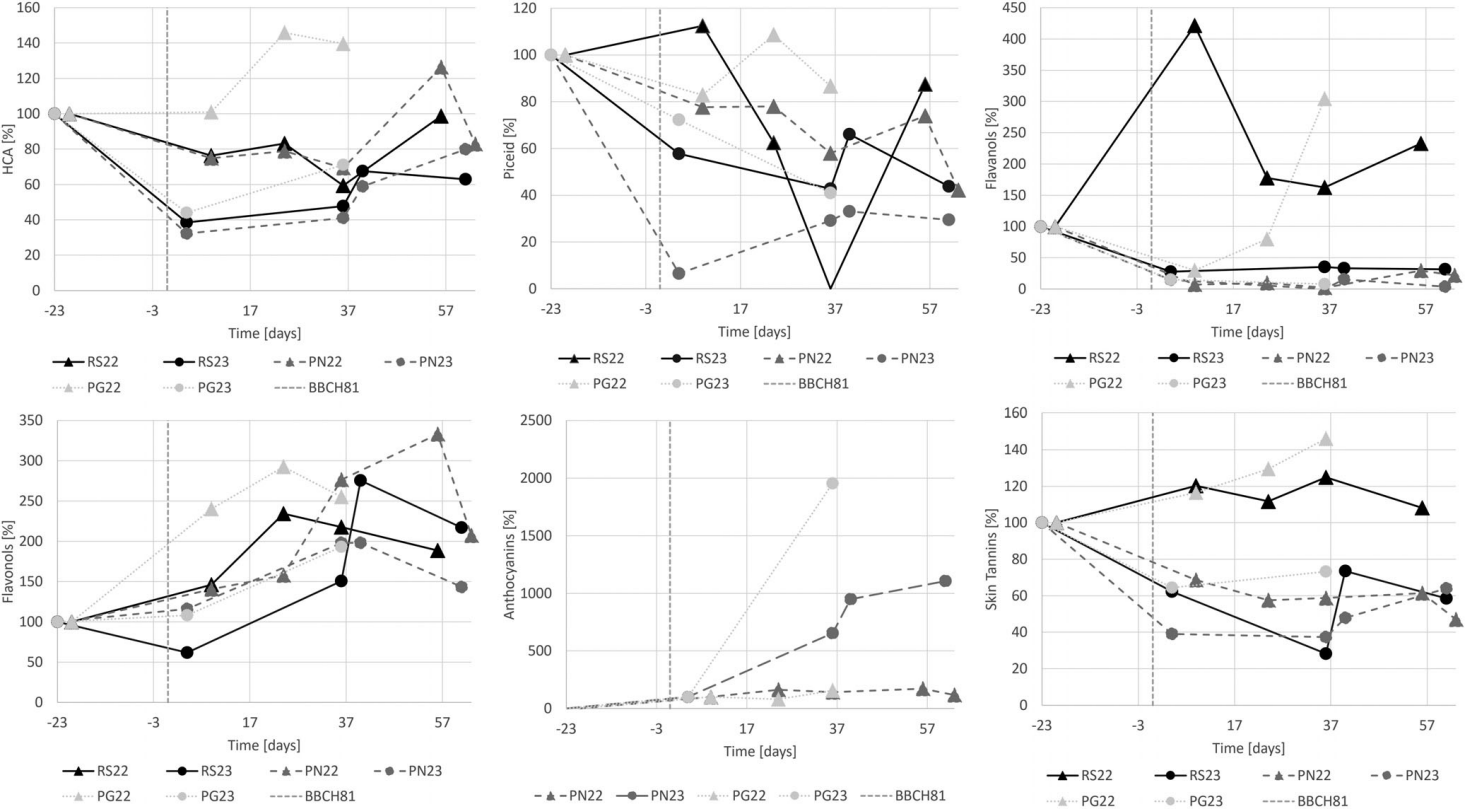
Phenolic content of the grape varieties Riesling (RS), Pinot gris (PG) and Pinot noir (PN) of two consecutive vintages (2022 and 2023) analyzed via UHPLC‐ESI‐MS^n^. Concentrations were normalized to their respective maximum values and expressed as percentages. HCA, hydroxycinnamic acids. BBCH value determined for Riesling.

HCA increased before (R and PN) or without (PG) infection, although decreases throughout ripening are generally expected.^31^ This pattern may reflect geographical conditions of the vineyards, as shown by Dal Santo *et al*.^43^ In RS, HCA decreases coincided with moderate BM in both vintages. In 2023, HCA losses occurred despite the absence of LA. In RS, HCA only increased at maximum BM and LA, whereas, in PN, increases appeared already at low BM and LA, followed by decreases as infection progressed. Figure 4 compares the normalized concentration of fertaric acid isomer II (FA II) with total HCA, showing that FA II follows overall HCA trend in both infested varieties but with greater variability. Deviations were smaller in the non‐infested PN 2023 and PG.

**Figure 4:**
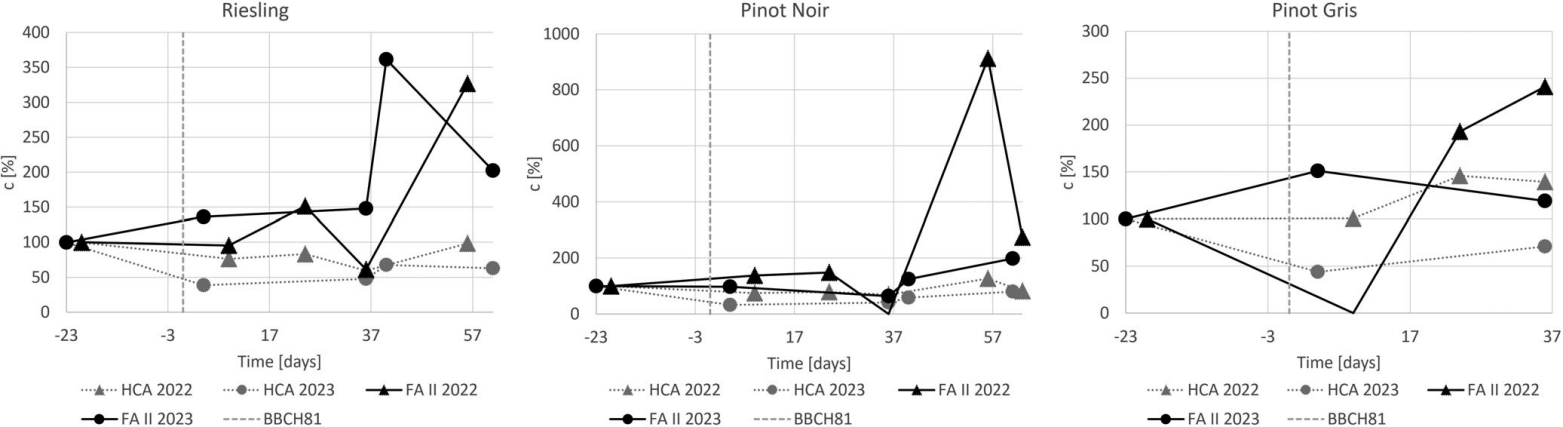
Hydroxycinnamic acid (HCA) and fertaric acid (FA II) content of the grape varieties Riesling (RS), Pinot gris (PG) and Pinot noir (PN) of two consecutive vintages (2022 and 2023) analyzed via UHPLC‐ESI‐MS^n^. Concentrations were normalized to their respective maximum values and expressed as percentages. BBCH value determined for Riesling.

Flavanol and piceid concentrations generally declined after BBCH 81. Without infection, levels remained mostly stable. Following infection, variety‐dependent differences were observed. In RS 2022, flavanols increased only at high BM and LA, whereas, in PN, increases occurred at significantly lower BM and LA. Flavanol concentrations were shown to be elevated as a result of *Botrytis* infections by Carbajal‐Ida *et al*.^22^ Stilbenes are generally considered to be increasingly produced during an infection, but the present results are inconsistent across varieties. In RS, piceid levels did not differ significantly between infested and healthy grapes, whereas, in PN, the expected increase was evident at the beginning of infection, followed by a decline as LA and BM rose, as shown by Jeandet *et al*.^20^

Anthocyanins increased steadily without biotic pressure, confirming the results reported by Nadal,^31^ and decreased after infection, consistent with Ky *et al*.^24^ Skin tannins peaked at sampling 1, immediately before the onset of ripening, in agreement with Gapinski *et al*.^44^ Ky *et al*.^24^ further described decreases after infection with *B. cinerea*, which can be confirmed here to varying extents.

Figure 5 displays the PCA for all three grape varieties classified by vintage, with samples containing detectable *Botrytis* biomass highlighted. The vintages differ markedly in vector orientation and sample distribution, whereas variety‐specific clustering is evident. Figure 6 comprises the PCA for the infested varieties RS and PN, respectively, in both vintages, which shows clear vintage‐dependent clustering. Flavonol vectors, and piceid in RS, are oriented perpendicular to other phenolic groups, suggesting a distinct behavior under grey mold pressure. Piceid in RS is the only variable near the *F*_2_ axis with an influence of roughly 31%. Pearson analysis confirmed significant positive correlations between LA and BM (*r* = 0.97), and between LA and HCA (*r* = 0.96).

**Figure 5:**
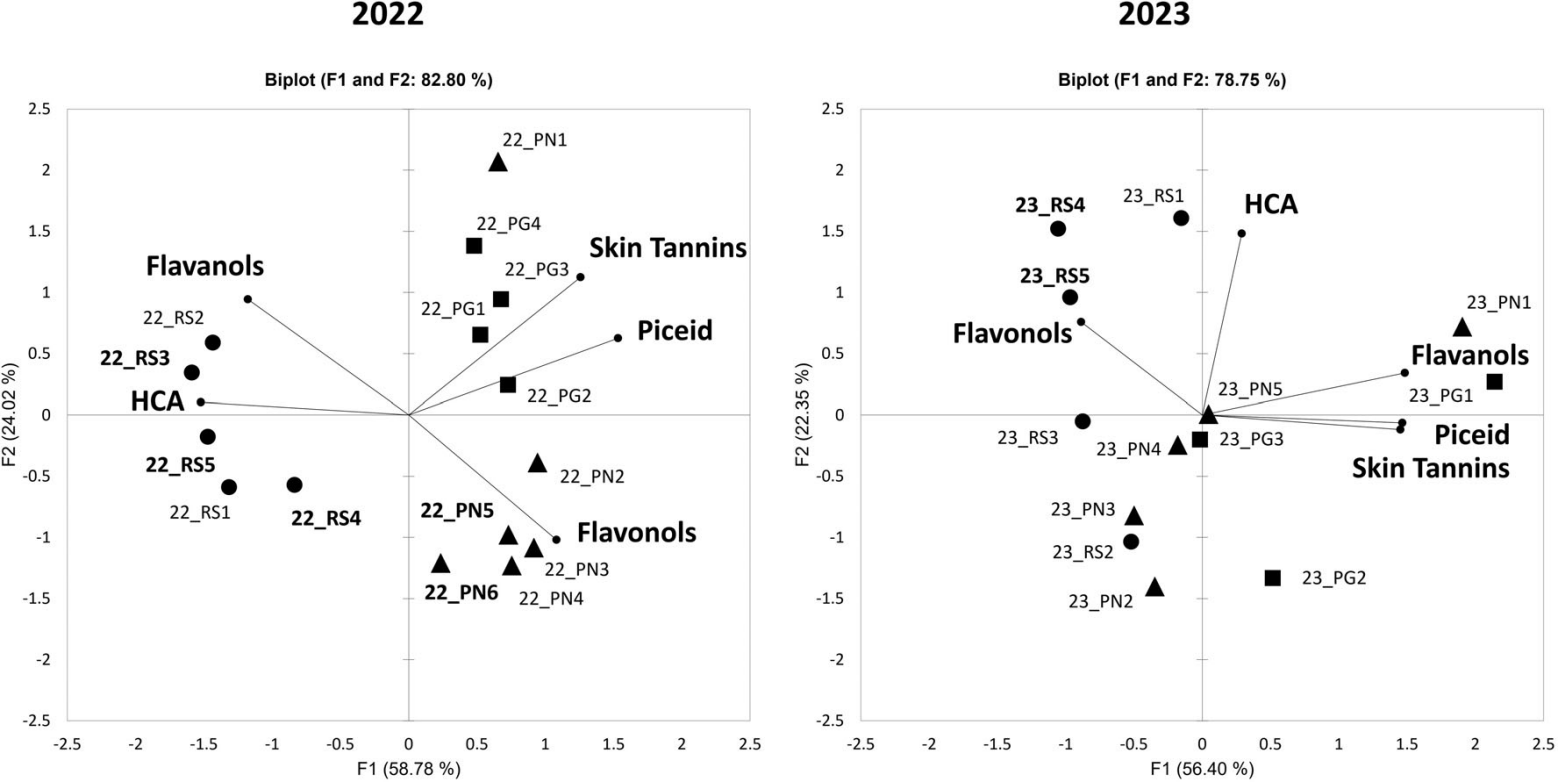
Principal component analysis (PCA) of phenolic groups in all three varieties (RS, Riesling; PG, Pinot gris; PN, Pinot noir) in 2022 (left) and 2023 (right). Samples with measurable infection parameters are marked bold. HCA, hydroxycinnamic acids.

**Figure 6:**
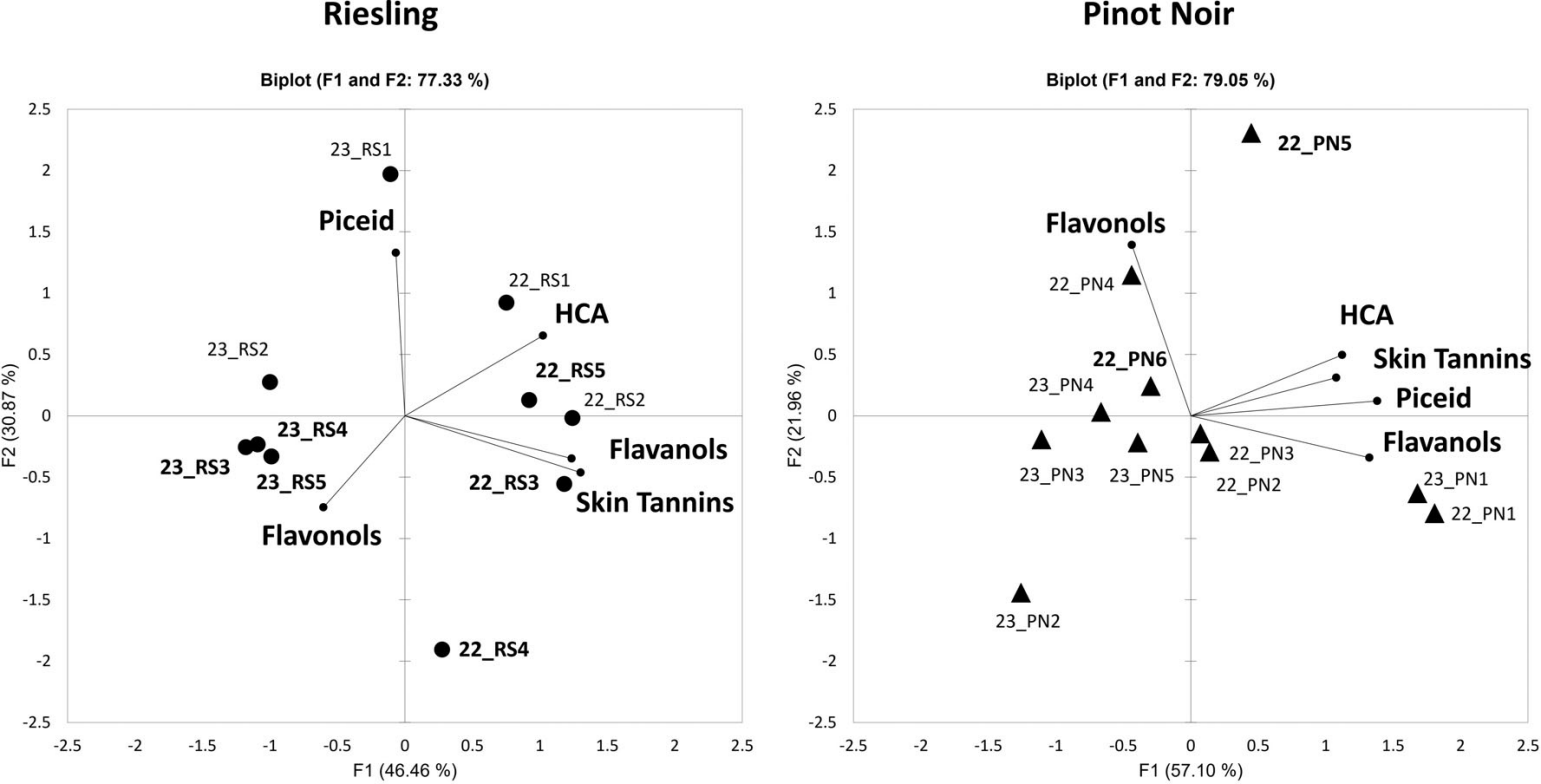
Principal component analysis (PCA) of phenolic groups in Riesling (RS, left) and Pinot noir (PN, right Samples with measurable infection parameters are marked bold. HCA, hydroxycinnamic acids.

### Substitution pattern of phenolic compounds throughout ripening

Figure 7 displays the phenolic content according to substitution patterns. The results were again normalized to their respective maximum, which was set to 100%. Most phenolic classes reach their maximum at véraison, which is in alignment with the role of polyphenols as phytoanticipins. Trihydroxylated compounds comprise only anthocyanins and the flavonol myricetin, and increase steadily. After véraison, the other groups (methoxylated, di‐ and monohydroxylated) increase also in the absence of infection. The infested grape varieties differ again because significant losses of methoxylated compounds can be observed already at moderate levels of BM in RS, without concomitant LA. Increases in methoxylated phenols are evident at high levels of BM and LA for this variety. By contrast, in PN, the concentration of methoxylated compounds increased already at low levels of BM and LA and decreased with proceeding infection. Mono‐ and trihydroxylated compounds exclusively decreased in PN and only at high BM and LA. Levels of dihydroxylated compounds decreased in both infested varieties. However, in RS, this reduction was observed regardless of the presence of LA. No increases are evident for mono‐, di‐ and trihydroxylated compounds. It is noteworthy that levels of trihydroxylated compounds are elevated for PG and PN compared to RS because of the presence of anthocyanins and higher levels of myricetin.

**Figure 7:**
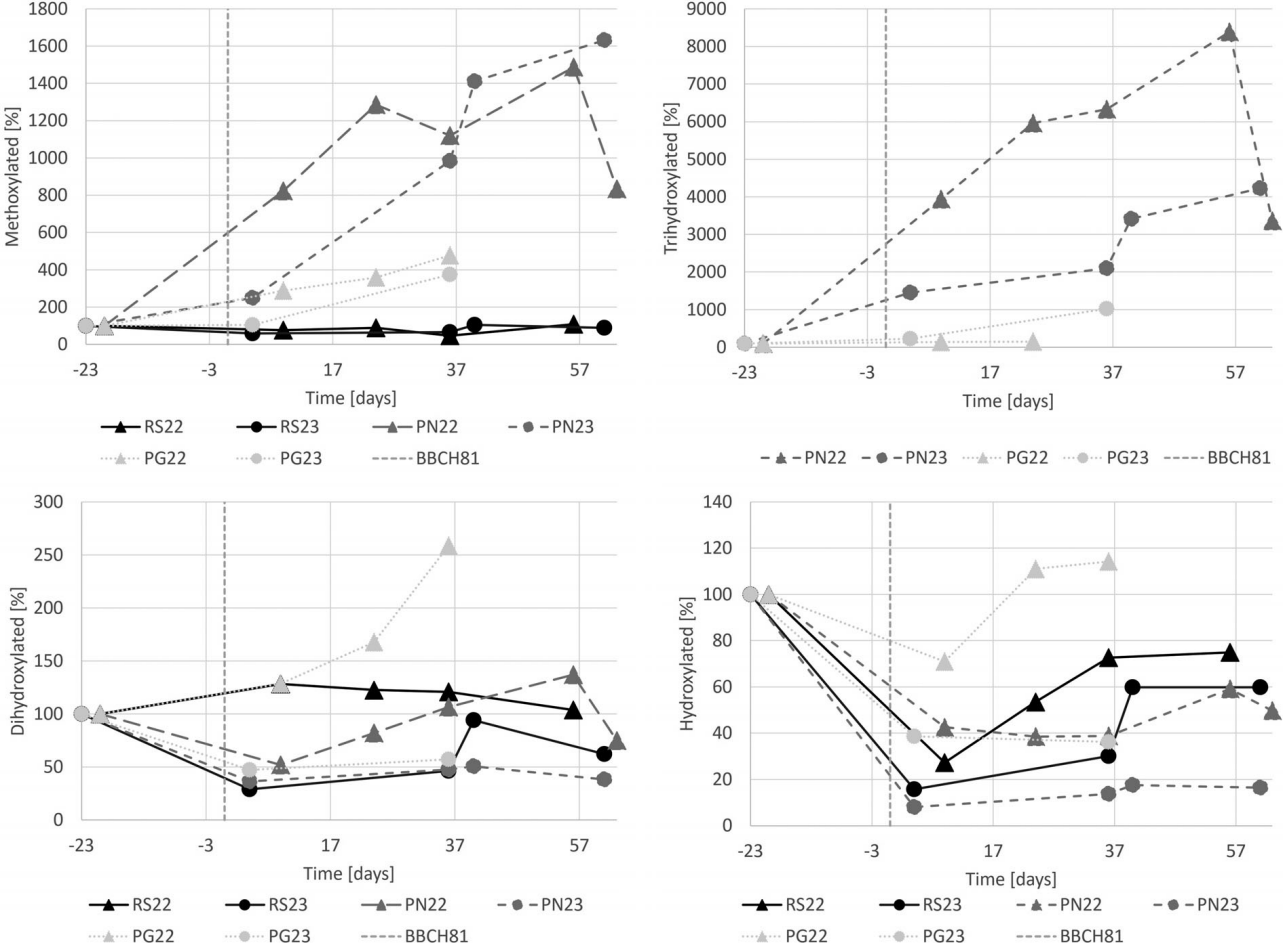
Phenolic content of the grape varieties Riesling (RS), Pinot gris (PG) and Pinot noir (PN) of two consecutive vintages (2022 and 2023) analyzed via UHPLC‐ESI‐MS^n^ and classified according to substitution patterns. Concentrations were normalized to their respective maximum values and expressed as percentages. BBCH value determined for Riesling.

## DISCUSSION

### Influence of precipitation on *B. cinerea* infection

The occurrence of BM and LA is likely a result of the substantial rainfall in 2022. This may be explained by elevated water uptake, resulting in larger berries and thus fruit cracking,^27^ which enables entry of the appressorium. The moderate rainfall observed in 2023 may account for the absence of infection in PN, as well as the delayed infection without accompanying laccase activity in RS. When assessing the risk of *Botrytis* infections, it is essential to consider not only microclimate and morphological grape features,^46^, ^47^ but also the degree of maturity.^48^ Comparing the amount of precipitation and TSS at the respective points in time shows variety‐specific differences in terms of susceptibility. In RS, which is presumed to be sensitive to *Botrytis* infections,^46^, ^47^ one spike in total rainfall with > 50 mm in 2022 and > 40 mm in 2023 after several weeks without precipitation led to infection in both vintages, probably because of more rapid water intake of berries. At the point of heavier rainfall, the TSS content was 13–14 °Bx. This appears to be a critical level for *Botrytis* infections, especially when heavy rainfall occurred after a long period of dryness. TSS levels increased to 16–18 °Bx by the time *Botrytis* biomass was detectable, approximately 2 weeks after the rainfall event each year. By contrast, PN was infested only after several heavy rainfall events in 2022 and at a much higher TSS content of > 21.6 °Bx. Rainfall events occurring between flowering and BBCH 81 may not significantly affect the infection process. This was evident in 2023, when only RS was infested, with infection occurring later during the ripening stage. Consequently, there appears to be a link between TSS content during heavy rainfall and the risk of infection, although the threshold varies greatly between grape varieties. These results corroborate the hypothesis regarding variety‐specific differences in susceptibility, indicating that RS exhibits a greater sensitivity to infections compared to PN.

### Phenolic profile in the context of vine stress response

Although establishing an average phenolic profile for a specific grape variety would be beneficial, achieving this is challenging because of the numerous factors that influence the phenolic composition of grape berries.^8^ As a result, it is even more difficult to draw conclusions about infection‐related changes. The non‐infested variety PG can serve as a basis to depict profile changes during ripening, and thus enable estimation of infection induced alteration of these profiles changes because of the close genotypic relation between the varieties. However, cultivar‐specific differences in phenolic profiles need to be taken into account. In PG, HCA steadily increase, which confirms the hypothesis that the phenylpropanoid and flavonoid metabolisms might be sensitive to pedoclimatic conditions, leading to constant increases in HCA in plain vineyards.^43^ Flavanol, piceid and anthocyanin trends emphasize the common understanding of phenolic synthesis without biotic stress. This comprises a significant drop after véraison and stagnation or increasing concentration in terms of anthocyanins due to continuous expression of flavonoid 3′‐hydroxylase (F3'H) and flavonoid 3′,5′‐hydroxylase (F3'5'H) during maturity.^38^ The development of tannins during the ripening process on the other hand is a complex issue,^49^ which limits the interpretation of the present results. In the non‐infested PG, skin tannins increase steadily, possibly as a result of sufficient expression of enzymes involved in the flavonoid pathway.

In the infested samples of RS and PN, variety‐specific changes in the phenolic profile are evident. This does not include the group of flavonols, which steadily increases. Most probably, the slight decrease in flavonols at the last sampling point cannot be attributed to infection because this phenomenon also occurred in the non‐infested samples. The prominent role of flavonols is underlined by its outstanding vector position in the PCA of the infested varieties. Consequently, flavonols seem to be the only phenolic group that is not affected by grey mold due to the absence of connections between BM, LA and flavonol levels.

In RS, levels of important phenolic compounds are reduced in early ripening stages, and the respective biosynthetic pathways for phytoalexins may only be induced at advanced levels of infection. Accordingly, certain branches of the flavonoid pathway may be up‐regulated. The occurrence of myricetin hexoside suggests the expression of F3'5'H as a response to *Botrytis* infection, which is usually not present in white grapes because of the remarkably low expression of the encoding genes.^38^ The possibility of enhanced F3'5'H expression in response to *B. cinerea* infection is supported by the observations by Blanco‐Ulate et al.^28^ regarding the induced expression of transcriptional regulators typical for red cultivars in noble rotten white berries. The absence of enhanced F3'5'H expression in noble rot berries noted by the latter suggests that this enzyme could be a key difference between noble rot and grey mold infections.

PN exhibits notable differences in its response to infection compared to RS because piceid, HCA and flavanols increased already at low levels of BM and LA. Therefore, key enzymes such as stilbene synthase (STS), phenylalanine ammonia lyase (PAL) or F3'H might have been induced earlier compared to RS. Losses in skin tannins, piceid and HCA observed at higher levels of BM and LA are more pronounced in PN than in RS. Consequently, changes in phenolic concentrations in the present data may not only be related to varying biosynthetic stress responses, but also dependent on the extent of infection in terms of BM and LA. Decreases following measurable LA might be attributed to laccase‐mediated oxidation, where substrate specificity differs tremendously between *B. cinerea* strains.^17^ In RS, low levels of BM and LA might already have led to decreases in phenolic concentrations. This might be a result of the hypothesized laccase‐mediated oxidation and/or a downregulation of biosynthetic pathways. Because no LA was detectable in samples harvested in 2023, losses can only be attributed to potentially attenuated biosynthesis.

In both varieties, the extent of phenolic losses differs between flavanols and HCA, which might be explained by a higher substrate specificity of the present laccases for HCA. Further investigation of the individual specificities is needed to draw conclusions here. A distinctive role can be assigned to fertaric acid because its losses are higher than for HCA in general. Ferulic acid, the main HCA in the grape berries examined here, is a particularly suitable substrate as a result of methoxylation,^50^ leaving it more susceptible to oxidative losses. The overall higher concentrations of HCA in RS compared to PN may act as a driving force for laccase secretion because HCA and especially ferulic acid possess laccase‐inducing properties.^16^ This may be an explanation for the observation that LA increases significantly faster in RS than in PN related to BM and underlines that BM and LA do not necessarily align.^16^ Thus, the increasing levels of HCA observed in both varieties may pave the way for proceeding infections. Ferulic acid poses the most important HCA here because its levels increase particularly in both infested varieties. This hypothesis is partly supported by the observations of Umberath *et al*.^16^ regarding growth‐inducing properties of HCA depending on the strain present. This contrasts with the findings of Patzke *et al*.^56^ on the growth‐inhibiting effects of ferulic acid. Consequently, the risk of accelerated infection by HCA is presumably dependent on the HCA profile and the *Botrytis* strain present. Concerning the increasing HCA levels, it is likely that the infection with *B. cinerea* induced up‐regulation of relevant enzymes in the HCA biosynthetic pathway such as PAL^21^, ^51^ or cinnamate 4‐hydroxylase (C4H).^28^ It needs to be emphasized that the latter study analyzed noble rotten berries, which evidentially differ from grey mold‐infested grapes in terms of transcriptional regulations.^23^ The assumption that HCA play a crucial role in the response of the grapevine to *B. cinerea* is supported by the significant Pearson correlation between both infection parameters and HCA.

Classifying phenols by substitution patterns can group together compounds from different metabolic pathways that would otherwise belong to separate subclasses. Because of the steady increase observed in non‐infested samples, it can be deduced that classification according to substitution pattern can provide a suitable basis for comparison with infected berries. In those, losses occur at various levels of BM and LA. Because the specificity of laccase is largely dependent on the substitution pattern, it can be hypothesized that the losses observed here may be a result of laccase‐mediated oxidation. Hence, substitution patterns of phenolic compounds may account for their susceptibility to laccase‐mediated losses and may therefore play an important role in the course of *Botrytis* infections. Levels of methoxylated phenols show the greatest variation following measurable BM and LA relative to the other substitutional classes, indicating their significance in the context of *Botrytis* infections. Increasing levels appear in PN at an earlier infection stage than in RS, which supports the aforementioned hypothesis of PN possibly modulating its phenolic profile more effectively than RS. Key enzymes involved in the synthesis of the methoxylated compounds quantified here involve PAL and *p*‐coumarate 3‐hydroxylase (C3H) (fertaric acid), F3'H (rhamnetin derivatives) and F3'5'H (syringetin derivatives and anthocyanins), for which potential up‐regulation may play a crucial role in the mutual influence of grapevine and fungus. The synthesis of methoxylated compounds further involves the expression of methyltransferases [e.g. *O*‐methyltransferase (OMT)]. Although past studies have shown that OMT play a role in stress‐induced regulatory mechanisms,^41^, ^52^ there is a lack of data on how grey mold can modulate genetic expression. The up‐regulation of caffeic acid 3‐*O*‐methyltransferase (COMT) observed by Blanco‐Ulate *et al*.^28^ during noble rot indicates possible modulation of OMT resulting from *B. cinerea* infections.

However, it is important to note that no gene expression analyses were conducted in the present study. Therefore, any drawn conclusions regarding the underlying causes of the observed effects are based on assumptions. In this context, it is also noteworthy that intervarietal differences could mask the effects of a *Botrytis* infection.

### Phenolic profile in the context of sensitivity

Differences in susceptibility to fungal infections can be attributed to morphological grape features such as cell wall composition or clustering,^27^ but the role modifications of phenolic profile play during infections remains unclear so far. The delayed increase in phenolic levels in RS may leave it very susceptible to (at least) this fungal disease. In addition, the increase in piceid observed here is significantly lower than expected,^20^, ^31^, ^45^ which suggests reduced up‐regulation of STS^21^ that may further contribute to its higher susceptibility. In comparison, a potentially more efficient up‐regulation of key enzymes of different synthetic pathways may contribute to the higher resistance of PN. This pattern appears in both conventional phenolic subgroups and classifications by substitution, supporting the hypothesis.

Another factor contributing to the greater resistance of PG and PN could be the content of trihydroxylated compounds. During ripening, a constant increase can be observed. This trend also applies when anthocyanins, which contribute significantly to the trihydroxylated content, are excluded. The remaining substance, myricetin, thus also shows steady increases regardless of infection, indicating that this group may not be negatively affected by *Botrytis* infestation (i.e. that no losses are evident). The significantly higher levels in both varieties are a noteworthy difference compared to the susceptible RS, as well as the lower amount of skin tannins in RS. Antimicrobial properties of (grape seed) tannins are extensively studied in terms of growth inhibition^53^, ^54^ and laccase inhibition.^55^ The high gallate levels in these tannin preparations^54^, ^55^ suggest that trihydroxylated phenols may contribute to their inhibitory effects. The possibility of myricetin induction in RS would support the hypothesis that trihydroxylated compounds play a crucial role in the context of *Botrytis* infections.

## CONCLUSIONS

Mutual influences between grape and fungal physiology constitute a complex factor in terms of susceptibility to *B. cinerea* infections. Global parameters such as climate, vintage and grape variety, as well as infection‐related parameters such as *Botrytis* biomass, laccase activity and the *Botrytis* strain present, can modulate phenolic profiles. To assess the specific effects of *B. cinerea* infections on phenolic composition, it would be beneficial to examine each factor individually before considering the overall impact. This study showed that potential grey mold‐related effects on phenolic profiles may comprise cultivar specific modifications based on two *Botrytis* infested varieties, one non‐infested variety and two consecutive vintages. Laccase activity and substitution patterns of phenolic compounds appear to play a significant role here. In the susceptible variety RS, losses in phenolic content were evident at low levels of infection, and up‐regulation of relevant biosynthetic pathways appears to commence only at advanced infestation. By contrast, PN showed the reverse pattern. This finding poses an opportunity for directing future research to explore further the genetic expressions of grapevines during grey mold infections and the role of laccase activity in it. The data of the non‐infested samples revealed that trihydroxylated compounds might be a decisive factor regarding resistance to *Botrytis* infections. This insight may be applied to future breeding of grape varieties that also considers the phenolic profile as an additional factor for resistance. However, to conclude the distinct effects of a *Botrytis* infection on the phenolic profile and to dissect these from other factors, significantly larger data sets covering several vintages of the same vines are needed.

## FUNDING

This IGF project of the FEI (Research Association of the German Food Industry) was supported within the programme of the German Federal Ministry of Economic Affairs and Energy on the basis of a decision by the German Bundestag (Project No. 01IF21630N).

## CONFLICTS OF INTEREST

The authors declare that they have no conflicts of interest.

## Supporting information


**Figure S1.** Hours of sunshine in both vintages and 10‐year average. Sampling dates and BBCH 81 are marked. BBCH value determined for Riesling.
**Table S1.** FTIR results for Riesling (RS), Pinot gris (PG) and Pinot noir (PN) in the Palatinate region during seasons 2022 and 2023. TSS, total soluble solids; TA, titratable acidity; D_81_, days prior to or after BBCH 81. BBCH value determined for Riesling.
**Table S2**. UHPLC‐ESI‐MS^n^ results for the phenolic profile of Riesling in vintages 2022 (left) and 2023 (right). Numbers in brackets represent [M‐H]^−^ of the precursor ion. n.d., not detected; n.q., not quantified. Quantification limits: resveratrol, 0.3 μg g^−1^; ferulic acid, 0.5 μg g^−1^; remaining phenols, 0.1 μg g^−1^. Detection limits: catechin, 0.04 μg g^−1^; caffeic acid: 0.01 μg g^−1^; ferulic acid, 0.2 μg g^−1^; *p‐*coumaric acid, 0.01 μg g^−1^; Q3G, 0.01 μg g^−1^; resveratrol, 0.1 μg g^−1^. Concentrations are expressed as μg g^−1^ of the respective equivalents, calculated per dry mass.
**Table S3.** UHPLC‐ESI‐MS^n^ results for the phenolic profile of Pinot gris in vintages 2022 (left) and 2023 (right). Numbers in brackets represent [M‐H]^−^ or, in case of anthocyanins, [M + H]^+^ of the precursor ion. n.d., not detected; n.q., not quantified. Quantification limits: resveratrol, 0.3 μg g^−1^; ferulic acid, 0.5 μg g^−1^; remaining phenols, 0.1 μg g^−1^. Detection limits: catechin, 0.04 μg g^−1^; caffeic acid: 0.01 μg g^−1^; ferulic acid, 0.2 μg g^−1^; *p‐*coumaric acid, 0.01 μg g^−1^; Q3G, 0.01 μg g^−1^; resveratrol, 0.1 μg g^−1^; M3G, 0.01 μg g^−1^. Concentrations are expressed as μg g^−1^ of the respective equivalents, calculated per dry mass.
**Table S4.** UHPLC‐ESI‐MS^n^ results for the phenolic profile of Pinot noir in vintages 2022 (left) and 2023 (right). Numbers in brackets represent [M‐H]^−^ or, in case of anthocyanins, [M + H]^+^ of the precursor ion. n.d., not detected; n.q., not quantified. Quantification limits: resveratrol, 0.3 μg g^−1^; ferulic acid, 0.5 μg g^−1^; remaining phenols, 0.1 μg g^−1^. Detection limits: catechin, 0.04 μg g^−1^; caffeic acid: 0.01 μg g^−1^; ferulic acid, 0.2 μg g^−1^; *p‐*coumaric acid, 0.01 μg g^−1^; Q3G, 0.01 μg g^−1^; resveratrol, 0.1 μg g^−1^; M3G, 0.01 μg g^−1^. Concentrations are expressed as μg g^−1^ of the respective equivalents, calculated per dry mass.

## Data Availability

The data that support the findings of this study are available from the corresponding author upon reasonable request.
