# Supplementary material for: Phenolic profile of selected grape ( Vitis vinifera L.) varieties in the course of ripening and Botrytis cinerea infections
Source: J Sci Food Agric. 2026 Aug 5;106(15):9753–63. doi: 10.1002/jsfa.70959 (PMC13743394; doi:10.1002/jsfa.70959)
Supplement: Supplementary file 1 [file JSFA-106-9753-s001.docx]

**Appendix A: Supplementary Data**

**Figure A1** Hours of sunshine in both vintages and 10-year average. Sampling dates and BBCH 81 are marked. BBCH value determined for Riesling.

**Table A1** FT-IR results for Riesling (RS), Pinot gris (PG), and Pinot noir (PN) in the Palatinate region during seasons 2022 and 2023. TSS, total soluble solids; TA, titratable acidity; D_81_, days prior to or after BBCH 81. BBCH value determined for Riesling.

|  |  | **2022** | | |  | |  | |  | **2023** | | |  | | |
| --- | --- | --- | --- | --- | --- | --- | --- | --- | --- | --- | --- | --- | --- | --- | --- |
| **Sampling Date** | **D_81_** | **TSS [°Bx]** | | | **TA [g/L]** | | | | **D_81_** | **TSS [°Bx]** | | | **TA [g/L]** | | |
|  |  | **RS** | **PG** | **PN** | **RS** | **PG** | | **PN** |  | **RS** | **PG** | **PN** | **RS** | **PG** | **PN** |
| 1 | -20 | N/A | | | N/A | | | | -23 | N/A | | | N/A | | |
| 2 | 9 | 13.8 | 17.6 | 17.6 | 17.7 | 11.8 | | 13.6 | 4 | 13.4 | 14.0 | 14.2 | 19.4 | 13.5 | 15.2 |
| 3 | 24 | 17.8 | 20.0 | 21.2 | 10.4 | 7.4 | | 8.1 | 36 | 16.2 | 17.0 | 18.0 | 12.9 | 8.3 | 9.7 |
| 4 | 36 | 18.8 | N/A | 21.6 | 9.9 | N/A | | 7.3 | 40 | 19.0 | 20.0 | 22.2 | 9.2 | 7.5 | 8.0 |
| 5 | 56 | N/A | | | N/A | | | | 61 | N/A | | | N/A | | |
| 6 | 63 | N/A | | | N/A | | | |  |  | | |  | | |

**Table A2** UHPLC-ESI-MS^n^ results for the phenolic profile of Riesling in vintages 2022 (left) and 2023 (right). Numbers in brackets represent [M-H]^-^ of the precursor ion. n.d., not detected; n.q., not quantified. Quantification limits: resveratrol, 0.3 µg/g; ferulic acid, 0.5 µg/g; remaining phenols, 0.1 µg/g. Detection limits: catechin, 0.04 µg/g; caffeic acid: 0.01 µg/g; ferulic acid, 0.2 µg/g; *p-*coumaric acid, 0.01 µg/g; Q3G, 0.01 µg/g; resveratrol, 0.1 µg/g. Concentrations are expressed as µg/g of the respective equivalents, calculated per dry mass.

|  | **2022** | | | | | **2023** | | | | |
| --- | --- | --- | --- | --- | --- | --- | --- | --- | --- | --- |
| **Precursor / Sampling Date** | **1** | **2** | 3 | **4** | **5** | **1** | **2** | **3** | **4** | **5** |
| *p*-Coumaric acid ethylester (191) | 23.9 | 32.3 | 31.7 | 31.6 | 35.0 | 21.8 | 13.5 | 21.0 | 24.5 | 27.3 |
| Catechin (289) | 2.0 | 7.3 | 5.4 | 2.5 | 5.0 | 0.8 | 0.3 | 0.3 | 0.4 | 0.3 |
| Epicatechin (289) | 0.8 | 4.1 | 4.4 | 1.9 | 2.9 | 0.1 | 0.2 | 0.3 | 0.2 | 0.2 |
| Caftaric acid (311) | 7.8 | 1.2 | n.d. | n.d. | 1.1 | 24.0 | 1.3 | 0.4 | n.d. | 0.4 |
| Coutaric acid (295) | 10.8 | 0.7 | n.d. | n.d. | 0.7 | 16.1 | 0.9 | 0.2 | 0.5 | 0.4 |
| (Iso)Rhamnetin I (315) | 0.2 | 0.3 | 0.3 | 0.3 | 0.4 | 0.2 | 0.2 | 0.2 | 0.3 | 0.3 |
| (Iso)Rhamnetin II (315) | 0.1 | 0.4 | 0.6 | 0.6 | 0.7 | 0.1 | 0.4 | 0.8 | 1.0 | 1.0 |
| Fertaric acid I (325) | 62.6 | 44.6 | 48.2 | 28.2 | 39.9 | 42.7 | 22.8 | 23.5 | 31.4 | 34.5 |
| Fertaric acid II (325) | 11.2 | 10.7 | 17.0 | 6.8 | 36.5 | 4.9 | 6.6 | 7.2 | 17.6 | 9.9 |
| Piceid (389) | 0.8 | 0.9 | 0.5 | n.d. | 0.7 | 1.2 | 0.7 | 0.5 | 0.8 | 0.5 |
| Kaempferol hexoside I (447) | 0.2 | n.d. | 1.3 | 1.9 | 1.7 | n.d. | 0.4 | 1.4 | 2.8 | 2.1 |
| Kaempferol hexoside II (447) | 0.3 | 1.5 | 4.1 | 6.3 | 5.9 | n.d. | 1.0 | 4.5 | 8.2 | 7.4 |
| Quercetin-3-galactoside (463) | 0.4 | 0.9 | 1.4 | 1.9 | 1.8 | 0.4 | 0.7 | 1.7 | 2.6 | 2.4 |
| Quercetin-3-glucoside (463) | 1.2 | 3.4 | 6.1 | 10.0 | 8.3 | 1.2 | 2.3 | 6.0 | 17.5 | 11.5 |
| Quercetin-3-glucuronide (477) | 9.3 | 10.4 | 5.9 | 7.6 | 4.4 | 11.0 | 7.0 | 6.7 | 12.5 | 5.8 |
| Myricetin hexoside I (479) | n.q. | n.q. | n.q. | n.d. | n.q. | n.q. | n.d. | n.q. | n.d. | n.d. |
| Myricetin hexoside II (479) | n.d. | n.d. | n.d. | n.d. | n.d. | n.d. | n.d. | n.d. | n.d. | n.d. |
| Procyanidin B-Type I (577) | 0.2 | 0.4 | 0.4 | 0.6 | 0.5 | 0.2 | n.d. | n.d. | n.d. | n.d. |
| Procyanidin B-Type II (577) | 0.1 | 0.5 | 0.5 | 0.7 | 0.5 | 0.1 | n.d. | n.d. | n.d. | n.d. |
| Procyanidin B-Type III (577) | n.d. | 0.4 | 0.6 | 0.8 | 0.5 | n.d. | n.d. | n.d. | n.d. | n.d. |
| Procyanidin B-Type IV (577) | n.d. | 0.2 | 0.4 | 0.5 | 0.4 | n.d. | n.d. | n.d. | n.d. | n.d. |
| Kaempferol-(rhamnosyl)hexoside (593) | 0.3 | 0.5 | 0.5 | 0.5 | 0.3 | 0.3 | 0.7 | 0.6 | 0.9 | 0.6 |
| Rutin (609) | 1.2 | 2.0 | 1.6 | 1.5 | 1.1 | 2.0 | 2.0 | 2.0 | 2.7 | 1.6 |
| GRP (Grape Reaction Product) (616) | n.q. | n.q. | n.q. | n.d. | n.q. | n.q. | n.q. | n.d. | n.q. | n.q. |
| Dihydrokaempferol pentoside I (433) | n.d. | n.d. | n.d. | 0.2 | 0.2 | n.d. | n.d. | n.d. | n.d. | n.d. |
| Dihydrokaempferol pentoside II (433) | n.d. | n.d. | n.d. | n.d. | n.d. | n.d. | n.d. | 0.1 | 0.2 | 0.2 |
| Dihydrokaempferol pentoside III (433) | n.d. | n.d. | n.d. | n.d. | n.d. | n.d. | n.q. | 0.1 | n.q. | n.d. |
| Dihydroquercetin rhamnoside (449) | n.d. | n.d. | n.d. | n.d. | n.d. | n.d. | n.d. | n.d. | n.d. | n.d. |
| Dihydroquercetin-3-glucoside (465) | n.d. | n.d. | n.d. | n.d. | n.d. | n.d. | n.d. | n.d. | n.d. | n.d. |
| Syringetin hexoside (507) | n.d. | n.d. | n.d. | n.d. | n.d. | n.d. | n.d. | n.d. | n.d. | n.d. |

**Table A3** UHPLC-ESI-MS^n^ results for the phenolic profile of Pinot gris in vintages 2022 (left) and 2023 (right). Numbers in brackets represent [M-H]^-^ or, in case of anthocyanins, [M+H]^+^ of the precursor ion. n.d., not detected; n.q., not quantified. Quantification limits: resveratrol, 0.3 µg/g; ferulic acid, 0.5 µg/g; remaining phenols, 0.1 µg/g. Detection limits: catechin, 0.04 µg/g; caffeic acid: 0.01 µg/g; ferulic acid, 0.2 µg/g; *p-*coumaric acid, 0.01 µg/g; Q3G, 0.01 µg/g; resveratrol, 0.1 µg/g; M3G, 0.01 µg/g. Concentrations are expressed as µg/g of the respective equivalents, calculated per dry mass.

|  | **2022** | | | | **2023** | | |
| --- | --- | --- | --- | --- | --- | --- | --- |
| **Precursor / Sampling Date** | **1** | **2** | **3** | **4** | **1** | **2** | **3** |
| *p*-Coumaric acid ethylester (191) | 31.8 | 47.0 | 52.3 | 60.5 | 21.1 | 19.0 | 31.9 |
| Catechin (289) | 2.3 | 0.6 | 2.5 | 8.3 | 5.1 | 0.6 | 0.3 |
| Epicatechin (289) | 0.4 | 0.8 | 0.6 | 0.6 | 0.3 | 0.2 | n.d. |
| Caftaric acid (311) | 3.4 | n.d. | n.d. | n.d. | 16.6 | 0.7 | n.d. |
| Coutaric acid (295) | 5.4 | n.d. | n.d. | n.d. | 15.9 | 0.5 | n.d. |
| (Iso)Rhamnetin I (315) | 0.1 | 0.3 | 0.3 | 0.3 | 0.1 | 0.2 | 0.2 |
| (Iso)Rhamnetin II (315) | 0.2 | 0.6 | 0.7 | 0.9 | 0.1 | 0.6 | 1.0 |
| Fertaric acid I (325) | 12.4 | 3.0 | 16.4 | 4.6 | 9.2 | 7.0 | 9.2 |
| Fertaric acid II (325) | 1.7 | n.d. | 3.3 | 4.2 | 1.9 | 2.9 | 2.3 |
| Piceid (389) | 3.2 | 2.7 | 3.5 | 2.8 | 6.9 | 5.0 | 2.8 |
| Kaempferol hexoside I (447) | n.d. | 0.5 | 1.2 | n.d. | n.d. | n.d. | n.d. |
| Kaempferol hexoside II (447) | 0.3 | 2.0 | 4.3 | 5.1 | n.d. | 1.3 | 2.7 |
| Quercetin-3-galactoside (463) | 0.3 | 1.4 | n.d. | n.d. | 0.5 | 0.8 | 1.8 |
| Quercetin-3-glucoside (463) | 1.5 | 11.1 | 16.0 | 25.9 | 2.0 | 4.3 | 9.0 |
| Quercetin-3-glucuronide (477) | 8.1 | 9.9 | 7.2 | 12.1 | 10.0 | 10.8 | 7.7 |
| Myricetin hexoside I (479) | n.q. | 1.1 | 1.8 | 2.6 | 0.1 | 0.1 | 0.8 |
| Myricetin hexoside II (479) | n.d. | 0.2 | 0.3 | 0.5 | n.d. | 0.1 | 0.2 |
| Procyanidin B-Type I (577) | 0.1 | n.d. | 0.5 | 0.3 | 0.9 | 0.4 | 0.3 |
| Procyanidin B-Type II (577) | n.d. | n.d. | n.d. | n.d. | 0.2 | n.d. | n.d. |
| Procyanidin B-Type III (577) | n.d. | n.d. | n.d. | n.d. | n.d. | n.d. | n.d. |
| Procyanidin B-Type IV (577) | 0.1 | n.d. | n.d. | n.d. | n.d. | n.d. | n.d. |
| Kaempferol-(rhamnosyl)hexoside (593) | 0.9 | 0.2 | n.d. | 0.3 | 0.1 | 0.3 | 0.3 |
| Rutin (609) | n.d. | 1.2 | 1.2 | 1.6 | 1.4 | 1.9 | 1.5 |
| GRP (Grape Reaction Product) (616) | n.q. | n.q. | 0.3 | n.q. | n.q. | 0.2 | 0.3 |
| Dihydrokaempferol pentoside I (433) | 0.1 | 0.5 | n.d. | n.d. | n.d. | n.d. | n.d. |
| Dihydrokaempferol pentoside II (433) | 0.3 | 1.8 | 2.7 | 3.8 | 0.1 | 0.3 | 0.4 |
| Dihydrokaempferol pentoside III (433) | 0.3 | n.d. | n.d. | n.d. | 0.2 | 1.4 | 2.3 |
| Dihydroquercetin rhamnoside (449) | n.d. | 0.3 | 0.4 | 0.5 | n.d. | 0.2 | 0.3 |
| Dihydroquercetin-3-glucoside (465) | n.d. | n.d. | n.d. | n.d. | n.d. | n.d. | n.d. |
| Syringetin hexoside (507) | 0.5 | 0.4 | 0.6 | 1.0 | n.d. | n.d. | 0.3 |
| Cyanidin-3-O-glucoside (449) | n.d. | n.d. | n.d. | n.d. | n.d. | n.d. | n.d. |
| Peonidin-3-O-glucoside (463) | n.d. | 8.5 | 7.5 | 9.3 | n.d. | n.d. | 3.0 |
| Delphinidin-3-O-glucoside (465) | n.d. | 0.8 | 0.8 | 1.2 | n.d. | n.q. | 0.4 |
| Petunidin-3-O-glucoside (479) | n.d. | 1.6 | 1.5 | 2.5 | n.d. | n.q. | 0.9 |
| Malvidin-3-O-glucoside (493) | n.d. | 29.8 | 27.5 | 51.1 | n.d. | 1.5 | 24.5 |

**Table A4** UHPLC-ESI-MS^n^ results for the phenolic profile of Pinot noir in vintages 2022 (left) and 2023 (right). Numbers in brackets represent [M-H]^-^ or, in case of anthocyanins, [M+H]^+^ of the precursor ion. n.d., not detected; n.q., not quantified. Quantification limits: resveratrol, 0.3 µg/g; ferulic acid, 0.5 µg/g; remaining phenols, 0.1 µg/g. Detection limits: catechin, 0.04 µg/g; caffeic acid: 0.01 µg/g; ferulic acid, 0.2 µg/g; *p-*coumaric acid, 0.01 µg/g; Q3G, 0.01 µg/g; resveratrol, 0.1 µg/g; M3G, 0.01 µg/g. Concentrations are expressed as µg/g of the respective equivalents, calculated per dry mass.

|  | **2022** | | | | | | **2023** | | | | |
| --- | --- | --- | --- | --- | --- | --- | --- | --- | --- | --- | --- |
| **Precursor / Sampling Date** | **1** | **2** | **3** | **4** | **5** | **6** | **1** | **2** | **3** | **4** | **5** |
| *p*-Coumaric acid ethylester (191) | 30.8 | 30.5 | 34.2 | 35.9 | 43.3 | 47.1 | 30.2 | 18.3 | 25.0 | 37.4 | 48.6 |
| Catechin (289) | 4.6 | 0.3 | 0.3 | 0.2 | 0.7 | 1.2 | 4.0 | 0.4 | n.d. | 0.5 | 0.3 |
| Epicatechin (289) | 1.0 | n.d. | 0.4 | n.d. | 1.9 | 0.7 | 0.4 | 0.2 | n.q. | 0.4 | n.q. |
| Caftaric acid (311) | 5.1 | n.d. | n.d. | n.d. | n.d. | n.d. | 21.1 | 0.6 | n.d. | n.d. | n.d. |
| Coutaric acid (295) | 10.1 | n.d. | n.d. | n.d. | n.d. | n.d. | 17.6 | 0.3 | n.d. | n.d. | n.d. |
| (Iso)Rhamnetin I (315) | 0.2 | 0.4 | 0.5 | 0.4 | 0.5 | 0.6 | 0.3 | 0.2 | 0.4 | 0.6 | 0.7 |
| (Iso)Rhamnetin II (315) | 0.2 | 0.5 | 0.6 | n.d. | 0.7 | 0.8 | 0.1 | 0.3 | 0.5 | 0.6 | 0.8 |
| Fertaric acid I (325) | 13.7 | 13.7 | 16.4 | 15.1 | 21.0 | 5.3 | 13.2 | 6.6 | 8.7 | 10.3 | 15.6 |
| Fertaric acid II (325) | 1.5 | 2.1 | 2.2 | n.d. | 13.8 | 4.1 | 2.6 | 2.6 | 1.7 | 3.3 | 5.2 |
| Piceid (389) | 3.7 | 2.9 | 2.9 | 2.1 | 2.7 | 1.6 | 4.6 | 0.3 | 1.4 | 1.5 | 1.4 |
| Kaempferol hexoside I (447) | n.d. | n.d. | n.d. | n.d. | 0.7 | 0.5 | n.d. | n.d. | n.d. | n.d. | n.d. |
| Kaempferol hexoside II (447) | n.d. | 1.4 | 1.5 | 1.9 | 2.6 | 1.9 | n.d. | 0.7 | 1.6 | 1.6 | 1.5 |
| Quercetin-3-galactoside (463) | n.d. | n.d. | 0.8 | n.d. | n.d. | n.d. | 0.4 | 0.7 | 1.0 | 1.0 | 1.0 |
| Quercetin-3-glucoside (463) | 1.9 | n.d. | 6.0 | 7.5 | 9.0 | 5.8 | 1.7 | 2.7 | 5.5 | 5.3 | 5.1 |
| Quercetin-3-glucuronide (477) | 7.2 | 9.1 | 7.2 | 11.7 | 12.1 | 5.4 | 6.9 | 5.8 | 7.0 | 6.7 | 8.2 |
| Myricetin hexoside I (479) | 0.1 | 1.8 | 2.5 | 2.8 | 3.3 | 1.4 | 0.1 | 0.5 | 0.9 | 0.9 | 1.1 |
| Myricetin hexoside II (479) | n.d. | 0.6 | 1.2 | 1.2 | 1.5 | 0.9 | n.d. | 0.2 | 0.8 | 1.2 | 1.3 |
| Procyanidin B-Type I (577) | 0.8 | 0.2 | 0.3 | n.d. | 0.2 | 0.2 | 0.8 | 0.3 | n.d. | n.d. | n.d. |
| Procyanidin B-Type II (577) | 0.3 | n.d. | n.d. | n.d. | n.d. | n.d. | 0.2 | n.d. | n.d. | n.d. | n.d. |
| Procyanidin B-Type III (577) | n.d. | n.d. | n.d. | n.d. | n.d. | n.d. | n.d. | n.d. | n.d. | n.d. | n.d. |
| Procyanidin B-Type IV (577) | n.d. | n.d. | n.d. | n.d. | n.d. | n.d. | n.d. | n.d. | n.d. | n.d. | n.d. |
| Kaempferol-(rhamnosyl)hexoside (593) | n.d. | n.d. | n.d. | n.d. | n.d. | 0.2 | 0.1 | 0.2 | 0.3 | 0.2 | 0.2 |
| Rutin (609) | 1.2 | n.d. | n.d. | n.d. | 1.6 | 1.0 | 1.3 | 1.4 | 1.4 | 1.1 | 1.0 |
| GRP (Grape Reaction Product) (616) | 0.1 | n.q. | n.q. | n.q. | 0.2 | n.q. | 0.1 | 0.1 | 0.1 | 0.2 | n.q. |
| Dihydrokaempferol pentoside I (433) | n.d. | n.d. | n.d. | n.d. | n.d. | 0.3 | 0.1 | 0.1 | 0.2 | n.d. | 0.3 |
| Dihydrokaempferol pentoside II (433) | n.d. | 1.1 | 1.4 | 1.6 | 1.9 | 2.0 | 0.1 | 0.4 | 0.9 | 1.1 | 1.4 |
| Dihydrokaempferol pentoside III (433) | n.d. | n.d. | 0.2 | n.d. | n.d. | 0.2 | n.d. | n.d. | n.d. | n.d. | 0.3 |
| Dihydroquercetin rhamnoside (449) | n.d. | 0.2 | 0.2 | 0.2 | 0.2 | 0.3 | n.d. | n.q. | n.q | n.q. | 0.2 |
| Dihydroquercetin-3-glucoside (465) | n.d. | 0.1 | 0.2 | 0.2 | 0.2 | 0.2 | n.d. | n.d. | 0.2 | 0.2 | 0.2 |
| Syringetin hexoside (507) | n.d. | n.d. | 1.4 | 1.9 | 2.2 | 1.5 | n.d. | 0.1 | 1.5 | 1.6 | 2.0 |
| Cyanidin-3-O-glucoside (449) | n.d. | 0.9 | 2.7 | 2.3 | 3.2 | 1.5 | n.d. | 0.3 | 0.7 | 1.8 | 2.0 |
| Peonidin-3-O-glucoside (463) | n.q. | 13.5 | 53.8 | 41.9 | 52.1 | 34.4 | n.d. | 5.1 | 27.9 | 65.5 | 73.8 |
| Delphinidin-3-O-glucoside (465) | n.d. | 2.7 | 3.9 | 4.1 | 6.0 | 2.0 | n.d. | 1.1 | 1.2 | 1.4 | 1.8 |
| Petunidin-3-O-glucoside (479) | n.d. | 5.6 | 8.2 | 8.1 | 10.7 | 4.4 | n.d. | 1.7 | 3.2 | 3.6 | 4.2 |
| Malvidin-3-O-glucoside (493) | n.q. | 89.0 | 112.1 | 104.1 | 129.4 | 83.6 | n.d. | 25.2 | 127.6 | 144.9 | 171.7 |
